# Captivity restructures the gut microbiota of François' langurs (*Trachypithecus francoisi*)

**DOI:** 10.3389/fmicb.2023.1166688

**Published:** 2023-05-12

**Authors:** Fengxiang Mo, Yuhui Li, Zheng Liu, Jingjin Zheng, Zhonghao Huang

**Affiliations:** ^1^Key Laboratory of Ecology and Endangered Species and Environmental Protection (Guangxi Normal University), Ministry of Education, Guilin, China; ^2^Guangxi Key Laboratory of Rare and Endangered Animal Ecology, Guangxi Normal University, Guilin, China; ^3^College of Life Sciences, Guangxi Normal University, Guilin, China

**Keywords:** gut microbiota, captivity, François' langurs (*Trachypithecus francoisi*), captive and wild, wild

## Abstract

Gut microbiota is crucial to primate survival. Data on the gut microbiota of captive and wild animals can provide a physiological and ecological basis for the conservation of rare and endangered species. To study the effect of captivity on the gut microbiota, we examine the difference in the gut microbiota composition between captive and wild Francois' langurs (*Trachypithecus francoisi*), using 16S rRNA sequencing technology. The results showed that the composition of the gut microbiota of captive and wild langurs was characterized by Firmicutes (51.93 ± 10.07% vs. 76.15 ± 8.37%) and Bacteroidetes (32.43 ± 10.00% vs. 4.82 ± 1.41%) at the phylum level and was characterized by Oscillospiraceae (15.80 ± 5.19% vs. 30.21 ± 4.87%) at the family level. The alpha diversity of gut microbiota in captive langurs was higher than those in wild, such as the Shannon index (4.45 ± 0.33 vs. 3.98 ± 0.19, *P* < 0.001) and *inv*Simpson index (35.11 ± 15.63 vs. 19.02 ± 4.87, *P* < 0.001). Principal coordinates analysis (PCoA) results showed significant differences in the composition of gut microbiota between captive and wild langurs at both the phylum and family levels (weight UniFrac algorithm, phylum level: *R*^2^ = 0.748, *P* = 0.001; family level: *R*^2^ = 0.685, *P* = 0.001). The relative abundance of Firmicutes (51.93 ± 10.07%) in captive langurs was lower than that of wild langurs (76.15 ± 8.37%), and the relative abundance of Bacteroidetes (32.43 ± 10.00%) in captive langurs was higher than that of wild (4.82 ± 1.41%). Our study concludes that dietary composition could be a crucial determinant in shaping the gut microbiota of langurs because more fiber-rich foods used by the wild langurs could increase the abundance of Firmicutes, and more simple carbohydrate-rich foods consumed by the captive langurs increase the abundance of Bacteroidetes. We highlight the importance of captivity on the gut microbiota and the need to consider the gut microbiota in animal provision.

## 1. Introduction

Animals' genetics and other factors have determined the influence on the gut microbiota (Goodrich et al., 2014, 2016; Bonder et al., 2016). The gut microbiota composition of non-human primates is predominantly composed of Firmicutes, Bacteroidetes, Verrucomicrobia, and Actinobacteria (Gomez et al., 2015; Hale et al., 2019; Chen et al., 2020b; Baniel et al., 2021), whereas that of reptiles, such as the Northern grass lizards (*Takydromus septentrionalis*), is mainly composed of Proteobacteria and Firmicutes (Zhou et al., 2020). Environmental factors are also essential for the gut microbiota composition (Sun et al., 2016; Orkin et al., 2019; Li et al., 2021). Commonly, food resources for most animals seasonally vary in accordance with climatic condition fluctuations (Lu et al., 2016). In response to the seasonal changes in foods, animals adjust their dietary compositions, consequently leading to seasonal variations in their gut microbiota. For instance, Tibetan macaques (*Macaca thibetana*) tend to eat more fiber-rich foods when foods are scarce in winter, resulting in an increase of the *Succinivibrio* to promote the digestion of cellulose and hemicellulose (Sun et al., 2016).

Diet also has a vital impact on gut microbiota composition and function (Rothschild et al., 2018; Huang et al., 2021; Jin et al., 2021). For example, animals that depended primarily on fibrous food, such as leaves, have a higher proportion of bacteria with the function of breaking down cellulose (Orkin et al., 2019; Sun et al., 2020). Moreover, animals that are dependent on a high-fat and low-fiber diet are rich in bacteria with the function of digesting simple carbohydrates and polysaccharides (Orkin et al., 2019; Sun et al., 2020). Due to the determined roles of diets in the gut microbiota, the host animals change the structures and functions of gut microbiota in response to dietary composition fluctuations (Muegge et al., 2011; Huang et al., 2021). These patterns occur in several spatiotemporal scales. For example, when food compositions vary by seasons, the gut microbiota of animals also experience seasonal changes (Lu et al., 2016; Orkin et al., 2019; Li et al., 2021; Xia et al., 2021). Specifically, the function of given taxa in the gut microbiota significantly differs, likely leading to adaptive changes in the gut microbiota to facilitate digesting and decomposing foods (Orkin et al., 2019; Xia et al., 2021). Geophagy is the normal behavior in most primates, allowing them to obtain minerals by licking rocks or eating soils (Pebsworth et al., 2012; Li et al., 2014). This behavior also could be linked to the nutrient and microbial supplementation of the hosts (Johns and Duquette, 1991; Krishnamani and Mahaney, 2000). Geophagy has been considered a potential vector for the introduction of environmental microbes into the animal guts (Borruso et al., 2021).

Captivity can cause animals to lose their native microbiota (Frankel et al., 2019). For example, captive white-throated woodrats (*Neotoma albigula*) retain more native microbiota when fed natural foods (Martínez-Mota et al., 2020); however, Bacteroidetes in their gut microbiota is reduced after provisioning them with a low-fiber diet (Sonnenburg et al., 2016), likely causing a decreased capacity to produce short-chain fatty acids (Colston and Jackson, 2016). Similarly, the gut microbiota diversity of wild langurs is higher than that of captive groups (Chen et al., 2020a), likely due to the fact that the dietary composition of wild animals tends to be more diverse than that of captive individuals (Nelson et al., 2013; McKenzie et al., 2017). This pattern could, at least partly, be caused by the decrease in dietary diversity in captive populations (Hale et al., 2019). However, the lower gut microbiota diversity in captive animals is species-specific dependent compared to the wild groups (Lee et al., 2019; Chen et al., 2020a). For example, the diversity of gut microbiota in wild Guizhou snub-nosed monkeys (*Rhinopithecus brelichi*) is higher than that of captive groups (Hale et al., 2019). However, gut microbiota diversity does not decrease in captive Japanese macaques (*Macaca fuscata*) (Lee et al., 2019). Similarly, the gut microbiota richness of captive rhesus macaques (*M. mulatta*) is higher than those of wild members, and the bacterial community structures significantly differ (Chen et al., 2020a).

Various gut microbiota significantly differs in functions (Flint et al., 2012). Among numerous gut microbiota taxa, Firmicutes have the capacity to encode energy metabolism-related enzymes, producing various digestive enzymes for the decomposition of lots of substances and facilitating the digestion of food in hosts (Flint et al., 2012; Rowland et al., 2018). Moreover, Bacteroidetes are characterized by fermenting carbohydrates and degrading plant-derived material and short-chain fatty acids (Colston and Jackson, 2016). Increased abundance of Firmicutes and decreased abundance of Bacteroidetes are associated with obesity in mice and humans (Murphy et al., 2010), which is correlated with the energy harvest of animals. Furthermore, Actinobacteria has been considered to contribute to degrading aromatic compounds in foods and promoting nutrient absorption in the host, whereas Verrucomicrobia has the ability to perform microbial hydrolysis of polysaccharides (Reid et al., 2011). Moreover, *Prevotella* plays an essential role in complex polysaccharide degradation and utilization (León-Mimila et al., 2018). Considering the variations in the functions of specific taxa in food digestion, animals adjust the composition and structure of their gut microbiota in response to various dietary compositions.

François' langurs are the flagship species of limestone forests, exclusively distributed in Guangxi, Guizhou, and Chongqing provinces of China and Northern Vietnam. Illegal hunting and habitat fragmentation have led to a population decline in wild langurs (Zhou and Huang, 2021). There are also a number of provisioned individuals living in captive conditions for the purpose of wild population recovery. Previous studies focus on the behavioral ecology of the François' langurs, investigating their adaptation to karst forests from the perspective of behavioral strategies, including dietary composition (Huang et al., 2010), nutrient content of foods (Liu et al., 2016), habitat use (Chen et al., 2019), and activity budget (Liu and Bhumpakphan, 2020). François' langurs are typical leaf-feeding primates (Huang et al., 2010). Leaves account for a high proportion of their diets with a marked preference for young leaves (Huang et al., 2010). Langurs also choose fruits, flowers, and other plant items (Duan et al., 2020). Captive langurs' dietary composition differs from wild individuals. Their foods contain rich simple carbohydrates. Although the composition of their gut microbiota has been studied (Duan et al., 2020), the effect of captivity on the gut microbiota of François' langurs remains unclear. In this study, we used 16s rRNA high-throughput sequencing to study the gut microbiota of captive and wild François' langurs to understand variations in the structures and functions of their gut microbiota. We describe the composition and diversity of captive and wild langurs and examine their variations in gut microbiota. We test the following predictions:

- Differences in dietary composition between captive and wild langurs are marked. Captive langurs consume more simple carbohydrate-rich and less fiber-rich foods than the wild langurs (Nijboer and Clauss, 2006; Huang et al., 2010; Chen et al., 2018). The gut microbiota changes when the dietary composition significantly varies (Orkin et al., 2019; Sun et al., 2020). Therefore, we predict that the relative abundance of bacteria related to the digestion of simple carbohydrates in captive langurs would be higher, and the relative abundance of bacteria related to the digestion of cellulose would be lower than in wild langurs.
- The dietary diversity of wild langurs is higher than captive langurs, and long-term captivity could lead to the loss of the primordial microbiome (Frankel et al., 2019). In general, the diversity of gut microbiota in wild animals is higher than that in captive groups (Nelson et al., 2013; McKenzie et al., 2017). Therefore, we predict that the gut microbiota diversity of wild langurs would be higher than captive langurs.

## 2. Methods

### 2.1. Study sites and fecal sample collection

In this study, fecal samples were collected from the Chongzuo White-Headed Langur National Nature Reserve, Guangxi province, China (107°16′53″-107°59′46″E, 22°10′ 43″-22°36′55″N) and the Wuzhou Langur Breeding Center, Guangxi province, China, during February 2019. The nature reserve is 400–600 m above sea level and is covered by karst limestone forests and is characterized by typical seasonal rainforests (Guangxi Forestry Department, 1993). Habitat fragmentation within the reserve is severe due to human activities (Huang et al., 2008). The Wuzhou Langur Breeding Center has the largest provisioned François' langur population in China, with more than 300 François' langurs provisioned in captivity (Shi et al., 2014).

Wild langurs are typically folivorous (Huang et al., 2010) and use cliffs as their sleeping sites (Chen et al., 2019). During the study period, these langurs predominantly consumed leaves (91.3% in the feeding record) and fruits (6.35%) (unpublished data). The wild langurs included two groups, group A with 12 individuals and group B with eight individuals. Group A consisted of one adult male, six adult females, two juveniles, one subadult, and two juveniles, whereas group B was composed of one adult male and seven adult females. We collected feces from wild langurs in the early morning after the langurs left their sleeping sites. All the feces were distributed within a distance of more than 2 m from each other. However, we could not conduct individual identification.

The captive langurs completely depended on provisioned foods, including cakes (24.73%), fruits (18.28%), kernels (3.23%), and leaves (53.76%). Captive langurs in this study were separately provisioned in three cages, including nine adult males and six adult females. Cages were divided into an inner and an outer chamber. The inner chamber has an enclosure of 4 m^*^2 m^*^3 m, whereas the outer chamber covered an enclosure of 4 m^*^ 4 m^*^4 m. The inner and outer chambers were connected by a window and a door. We collected samples from captive langurs in the morning when they defecated in the cage. During sample collection, we used sterile bamboo sticks to collect the fecal samples and chose the inner layer of fecal matter. We collected the samples in sterile 15 ml tubes, and then stored them in a dry icebox, immediately transferring all samples to ultra-low-temperature refrigerators in the laboratory and storing at −80°C until bacterial DNA extraction. We collected 32 samples, including 17 samples collected from wild langurs and 15 samples collected from captive langurs.

### 2.2. DNA extraction, 16S rRNA amplification, and sequencing

We chose the E.Z.N.A.^®^ Soil DNA kit (Omega Bio-Tek, Norcross, GA, USA) to extract bacterial DNA from all fecal samples. We conducted purification using a NanoDrop 2000 ultraviolet–visible light spectrophotometer (Thermo Fisher Scientific, Wilmington, DE, USA) and checked DNA quality by 1% agarose gel electrophoresis. We used universal bacterial primers (338F: 5'-ACTCCTACGGGAGGCAGCAG-3' and 806R: 5'-GGACTACHVGGGTWTCTAAT-3') to amplify the V3–V4 hyper-variable region of the 16S rRNA gene with a polymerase chain reaction (PCR) system (GeneAmp 9700, ABI, USA) (Mori et al., 2014). The PCR products were extracted from a 2% agarose gel, further purified by the AxyPrep DNA Gel Extraction kit (Axygen Biosciences, Union City, CA, USA), and quantified by QuantiFluor™-ST (Promega, USA). Purified amplified fragments were paired-end sequenced (2 × 300) on an Illumina MiSeq platform (Illumina, San Diego, CA, USA) by Majorbio Bio-Pharm Technology Co. Ltd (Shanghai, China).

### 2.3. Data analysis

We conducted an analysis based on the operational taxonomic units (OTU). We used a Silva138/16s_bacteria database for OTU clustering (Release138 http://www.arb-silva.de), using Uparse 7.0.1090 software (version 7.0.1090 http://drive5.com/uparse/). We extracted non-repetitive sequences from the optimized sequences, reduced the amount of redundant calculation in the process of analysis (http://drive5.com/usearch/manual/dereplication.html), and removed the single sequences without repetition (http://drive5.com/usearch/manual/singletons.html). OTU clustering was carried out for non-repeating sequences (excluding single sequences) according to 97% similarity, and chimeras were removed in the clustering process to obtain OTU representative sequences. We mapped all optimized sequences to OTU representative sequences, and then selected sequences with more than 97% similarity to OTU representative sequences, consequently generating an OTU table. Finally, taxonomic analysis was performed on the OTU tables generated by the RDP classifier Bayesian algorithm (version 2.11 http://sourceforge.net/projects/rdp-classifier/). The classification confidence was 0.8, and the community species composition of each sample was counted at the phylum level and family level.

When drawing the rarefaction curve, the abscissa represents the number of sequences selected from OTU sets, and the ordinate represents the alpha diversity index (the Shannon and *inv*Simpson index). The rarefaction curve reflects the variation trend of microbial species diversity in the samples with the increase in sequencing data volume. An asymptotic curve indicates that the sequencing depth is sufficient to reflect most of the biodiversity information of the samples (Amato et al., 2013). Community coverage was used to determine the quality of sequencing. A higher value of community coverage indicates a higher probability of sequence detection in the sample. Group samples of the community bar map were calculated as mean value, and the groups with relative abundance less than 0.01 were merged into “others”. To compare the difference in gut microbiota composition between the two groups, a Wilcoxon rank-sum test was used to carry out the two-tailed *t*-test at the phylum and family levels. *P*-values were corrected by multiple tests using false discovery rate adjustment, and the confidence interval was calculated using bootstrap of 0.95. The corrected *P*-values were used to indicate their significance.

Linear discriminant analysis effect size (LEfSe) multilevel species difference discriminant analysis was used to discover high-dimensional biomarkers and reveal genomic features (Segata et al., 2011). The Kruskal–Wallis (KW) rank-sum test was used to detect significant differences in the relative abundance of gut microbiota. In this study, the classification level ranged from the phylum level to the family level, and the first-level grouping was set as captive and wild langurs, with an LDA threshold of 3.5 and homogenization of abundance. One-against-all (less strict) was selected as the multi-group comparison strategy, that is, if there were differences between any two groups, the group was considered a differential species. Linear discriminant analysis (LDA) was also used to indicate the impact of each species' abundance on the differential effect.

In this study, the Shannon index and *inv*Simpson index were used to reflect community diversity, and the Ace and the Chao index were used to reflect the community richness of gut microbiota (Schloss et al., 2009). Principal coordinates analysis (PCoA) was used to confirm the similarity in the composition of gut microbiota between captive and wild langurs. Adonis was used for the difference test, with weighted and unweighted UniFrac used for the distance algorithm, and the number of substitutions was 999.

PICRUSt was used to standardize the OTU table to remove the influence of the copy number of the 16S marker gene in the species genome. Then, COG family information and KEGG Ortholog (KO) information corresponding to OTU were obtained by greengene id corresponding to each OTU (Kyoto Encyclopedia of Genes and Genomes, http://www.genome.jp/kegg/). Based on information from the KEGG database, Pathway 1 and Pathway 2 information can be obtained, and the abundance of each functional category can be calculated based on OTU abundance. Finally, a *t*-test was used to compare the difference in gut microbiota function profiles between the two groups.

## 3. Results

### 3.1. Sequence quality evaluation

**Figure 1:**
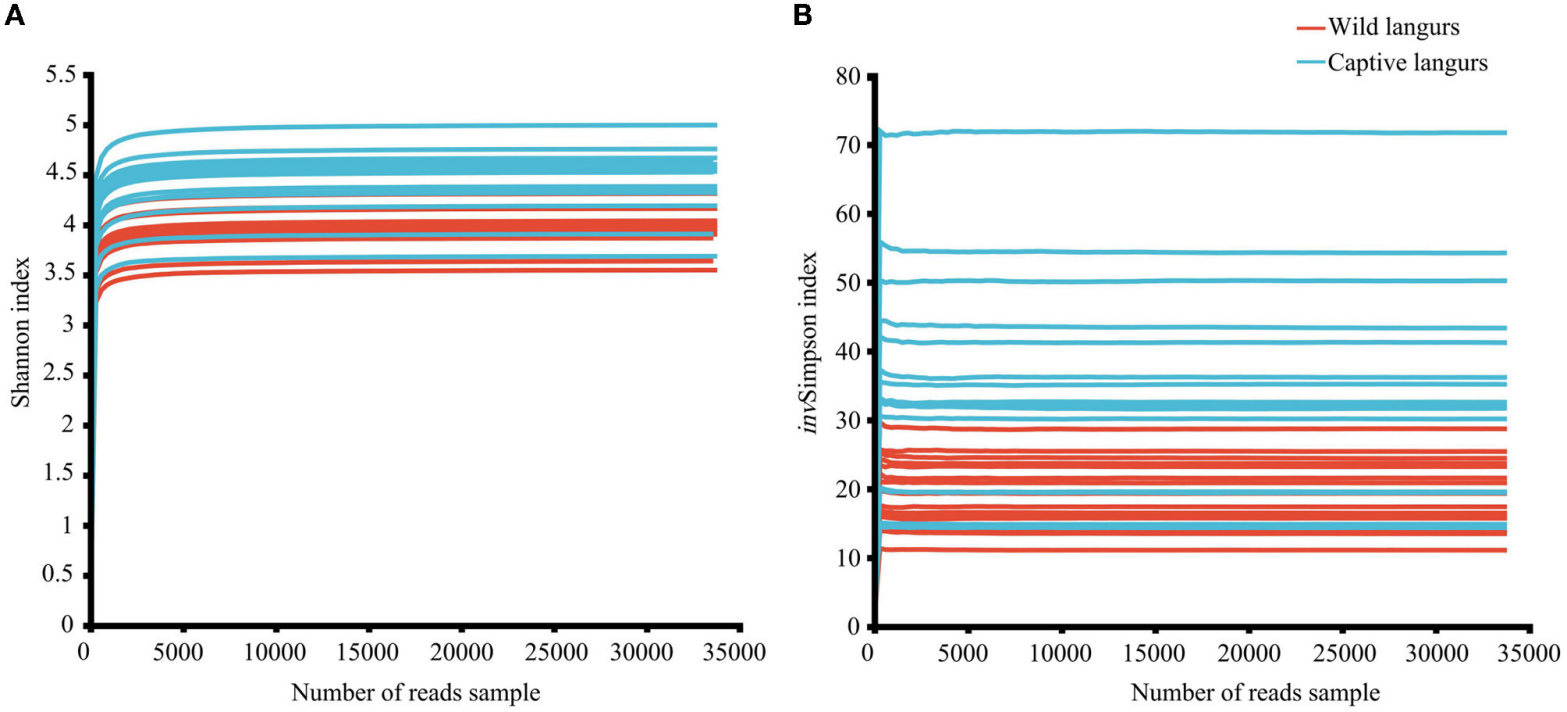
Rarefaction curves of Shannon **(A)** and invSimpson **(B)** index of the gut microbiota of François' langurs.

In this study, the total number of optimized sequences after two-terminal sequence quality control splicing was 2,005,306; the average number of sequences was 62,665.81 ± 7353.22, with a sequence length of 410.61 ± 4.10 bp. The species coverage of all samples ranged from 99.65% to 99.81%, indicating that the sequencing results sufficiently reflected the microbial species in the samples. All the rarefaction curves of the Shannon and *inv*Simpson index in the diversity dilution curve tended to be asymptotic eventually, indicating that the amount of sequencing data was sufficient to reflect the majority of microbial diversity information in the samples (Figure 1).

### 3.2. Variations in the gut microbiota composition between captive and wild langurs

**Figure 2:**
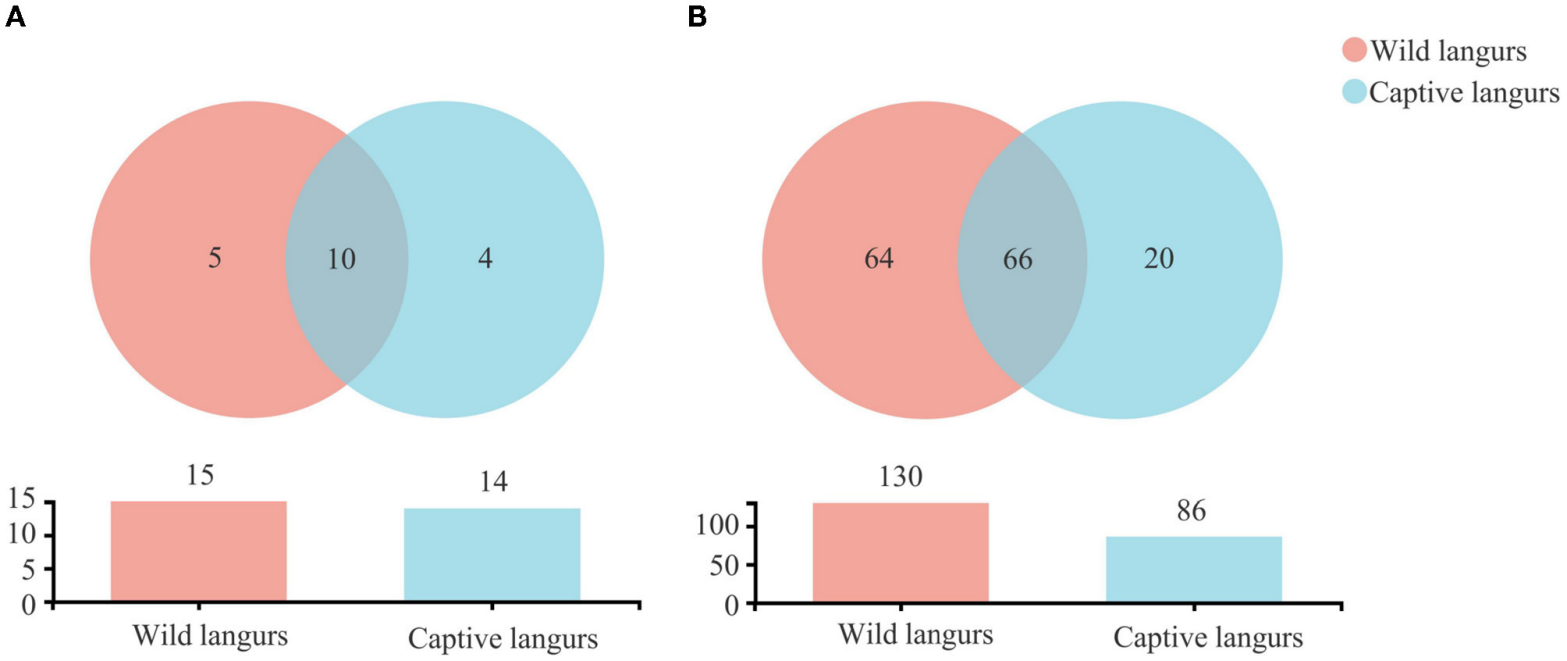
Shared and specific taxa in the gut microbiota of wild and captive François' langurs at the phylum **(A)** and family **(B)** levels.

There were 1,390 OTUs obtained from all samples, belonging to 19 phyla, 34 classes, 85 orders, 150 families, 294 genera, and 460 species (Figure 2). The gut microbiota of the captive langurs included 86 families of 14 phyla and 20 families of 4 phyla, whereas the gut microbiota of the wild langurs included 15 phyla, 130 families, 5 phyla, and 64 families. There were 10 phyla and 66 families shared by both groups.

**Figure 3:**
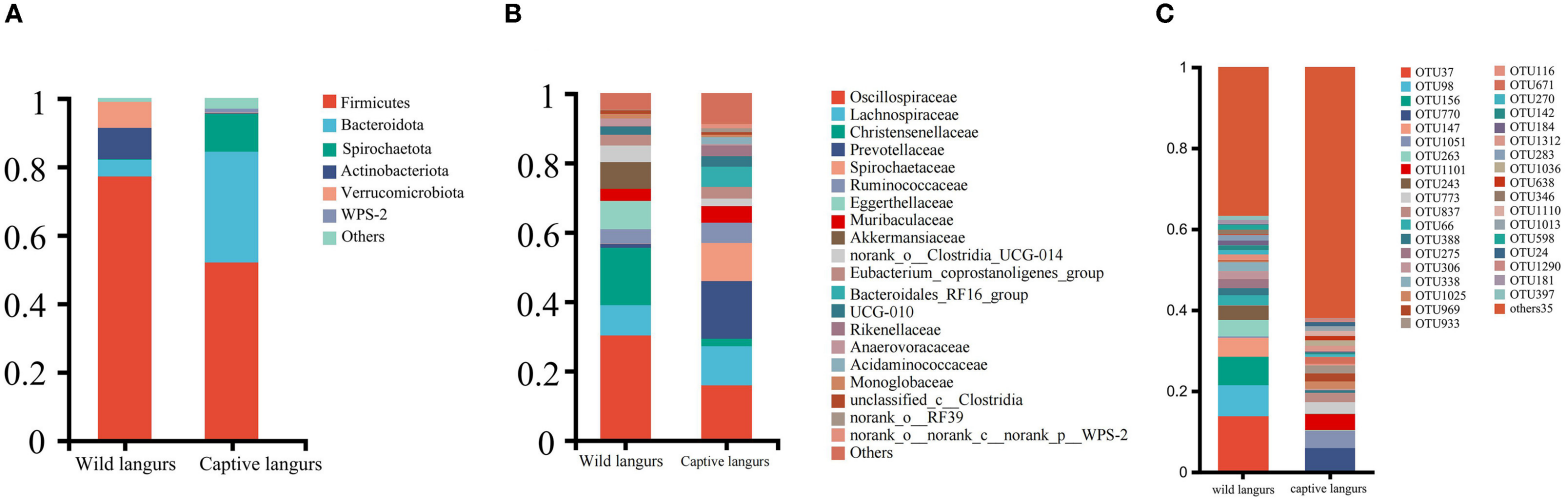
Community composition of the gut microbiota of François' langurs at the phylum **(A)**, family **(B)**, and OTU **(C)** levels (Others: taxa with less than 1% relative abundance).

At the phylum level, the gut microbiota of captive langurs was predominantly characterized by Firmicutes (51.93 ± 10.07%), Bacteroidetes (32.43 ± 10.00%), and Spirochaetes (11.04 ± 7.29%) (Figure 3A; Supplementary Informations 1, 3A). At the family level, the predominant bacteria families were Prevotellaceae (16.61 ± 12.20%), Oscillospiraceae (15.80 ± 5.19%), Lachnospiraceae (11.28 ± 4.16%), Spirochaetaceae (11.04 ± 7.29%), and Ruminococcaceae (5.79 ± 1.57%; Figure 3B; Supplementary Informations 2, 3B).

At the phylum level, the most frequently detected gut microbiota in wild langurs was Firmicutes (76.15 ± 8.37%), followed by Actinobacteria (9.11 ± 8.20%) and Verrucomicrobia (7.71 ± 5.52%; Figure 3A; Supplementary Informations 1, 3A). At the family level, the most frequently occurred taxon was Oscillospiraceae (30.21 ± 4.87%), followed by Christensenellaceae (16.56 ± 2.66%), Lachnospiraceae (8.71 ± 3.16%), Eggerthellaceae (8.24 ± 8.37%), and Ruminococcaceae (4.08 ± 2.81%; Figure 3B; Supplementary Informations 2, 3B).

At the OTU level, the top five OTUs in the relative abundance of gut microbiota in the wild langurs included OTU37 (13.74%, Oscillospiraceae), OTU98 (7.67%, Akkermansiaceae), OTU156 (7.04%, Oscillospiraceae), OTU147 (4.64%, Eggerthellaceae), and OTU263(4.08%, Christensenellaceae). The top five OTUs in the relative abundance consisted of OTU770 (5.74%, Spirochaetaceae), OTU1051 (4.26%, Oscillospiraceae), OTU1101 (3.93%, Prevotellaceae), OTU773 (2.83%, Bacteroidales_RF16_group), and OTU837 (2.36%, Oscillospiraceae) (Figure 3C; Supplementary Information 3C).

**Figure 4:**
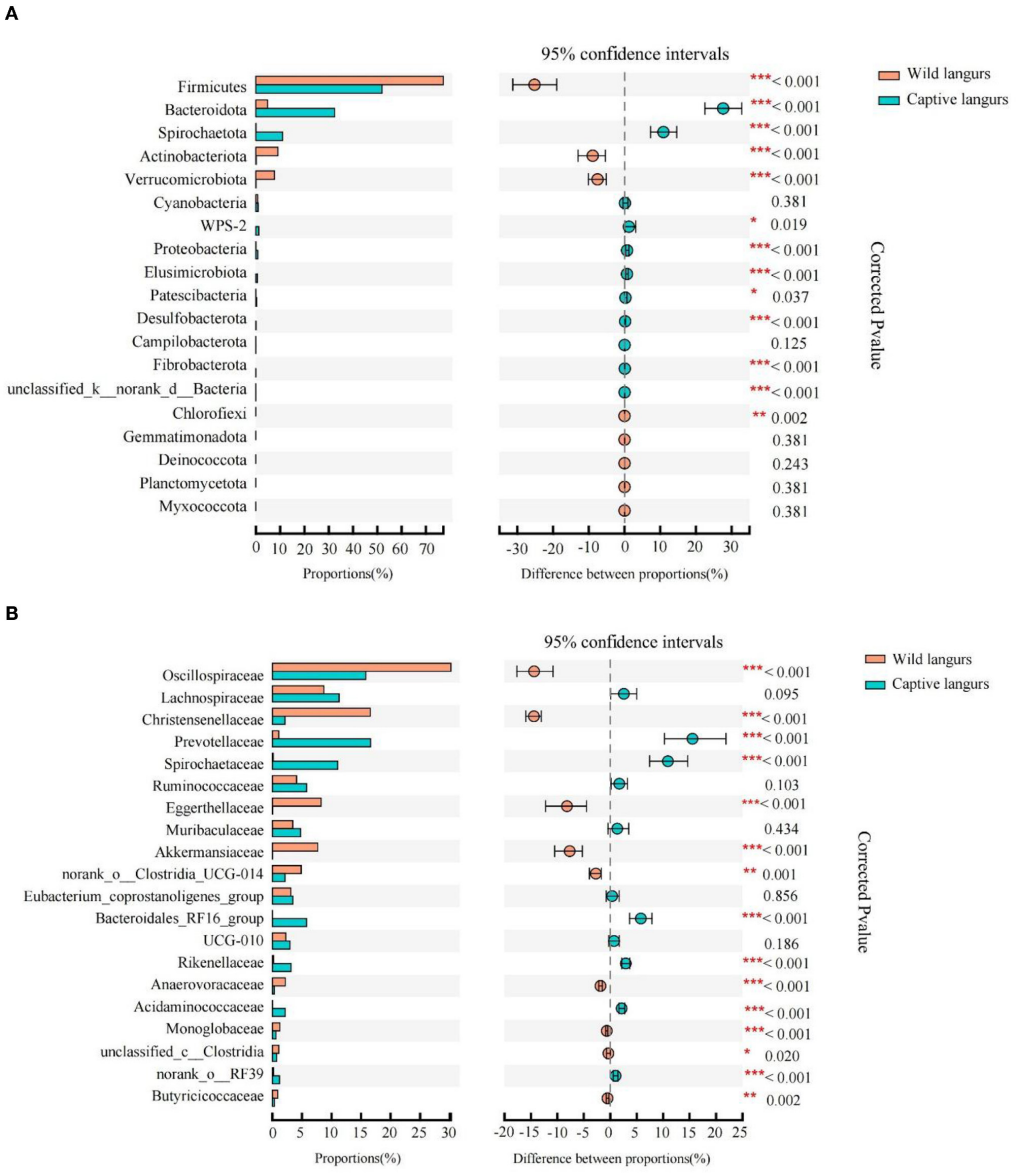
Difference in the relative abundance of gut microbiota of François' langurs [**(A)**: all taxa at the phylum level; **(B)**: top 20 taxa at the family level; the bar graph on the left side shows the relative abundance of taxa; the right side shows the statistics for seasonal differences in taxa; the color of circle shows the season with higher relative abundance].

According to Wilcoxon rank-sum tests, the proportion of gut microbiota significantly differed between the wild and captive langurs. At the phylum level, Bacteroidota, Spirochaetota, and Proteobacteria were more enriched in captive langurs, whereas Firmicutes, Actinobacteriota, and Verrucomicrobiota were more enriched in wild langurs (Figure 4A; Supplementary Information 1). At the family level, Prevotellaceae, Spirochactaceae, and Bacteroidales_RFI6_group were more enriched in captive langurs, whereas Oscillospiraceae, Christensenellaceae, and Eggerthellaceae were more enriched in wild langurs (Figure 4B; Supplementary Information 2).

**Figure 5:**
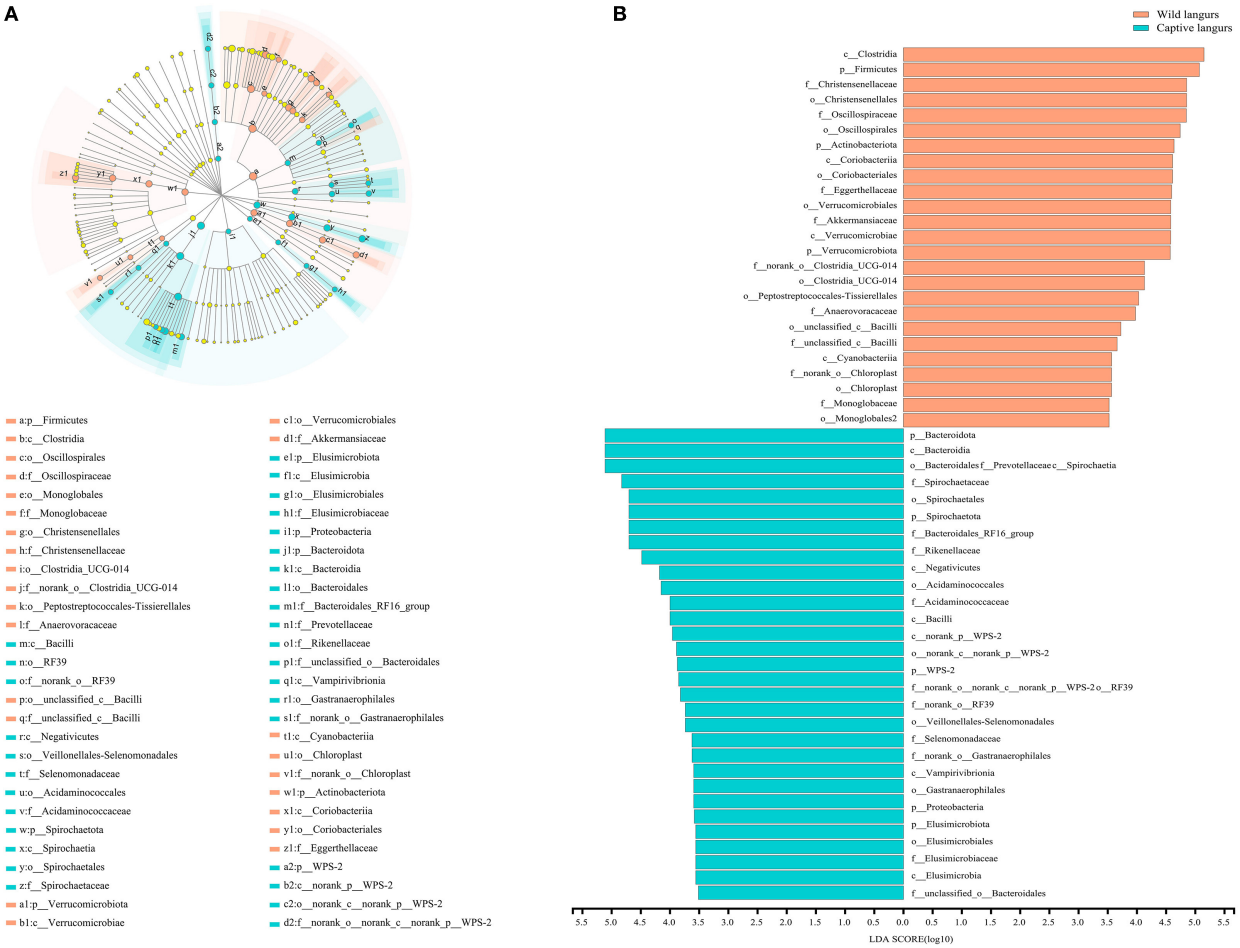
**(A)** Cladogram of LEfSe analysis for seasonal variations in the gut microbiota of François' langurs (the differences in relative abundance from the phylum to family levels, and the orange and light green circles indicate differences in relative abundance and yellow circles indicate non-significant differences). **(B)** LDA score histogram for differential taxa in the gut microbiota of François' langurs (the larger LDA scores indicate a greater influence of taxa on seasonal variations).

To further identify the shift in the gut microbiota composition between wild and captive langurs, we used LEfSe to show differences in the relative abundance of the bacterial taxa at the phylum, class, order, and family levels. The significant taxa with an LDA score >3.5 are illustrated in Figure 5. LEfSe result showed 48 significantly enriched taxa, and the significantly enriched taxa of captive langurs (29) were more than wild langurs (19). Particularly, the Bacteroidota (LDA = 5.13), Bacteroidia (LDA = 5.12), Spirochaetia (LDA = 5.12), Spirochaetaceae (LDA = 4.81), and Spirochaetales (LDA = 4.72) had higher relative abundance in captive langurs, whereas the Clostridia (LDA = 5.12), Firmicutes (LDA = 5.12), Christensenellaceae (LDA = 5.12), Christensenellales (LDA = 4.79), and Oscillospiraceae (LDA = 4.68) had higher relative abundance in wild langurs.

### 3.3. Variations in the gut microbiota diversity between captive and wild langurs

**Figure 6:**
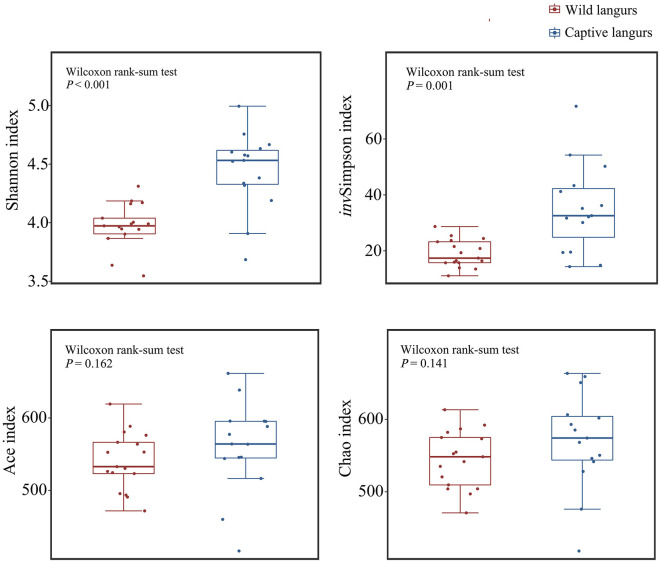
Difference in alpha diversity index in the gut microbiota of wild and captive François' langurs.

Alpha diversity analysis revealed that there was a significant difference in the Shannon (4.45 ± 0.33 vs. 3.98 ± 0.19) and *inv*Simpson indices (35.11 ± 15.63 vs. 19.02 ± 4.87) between captive and wild langurs; however, there were no significant differences in the Ace (560.56 ± 62.44 vs. 540.56 ± 39.72) and the Chao indices (570.86 ± 66.38 vs. 544.78 ± 39.52). These results indicated that there were differences in diversity and evenness between captive and wild langurs and indicated a similar richness for both langurs (Figure 6).

**Figure 7:**
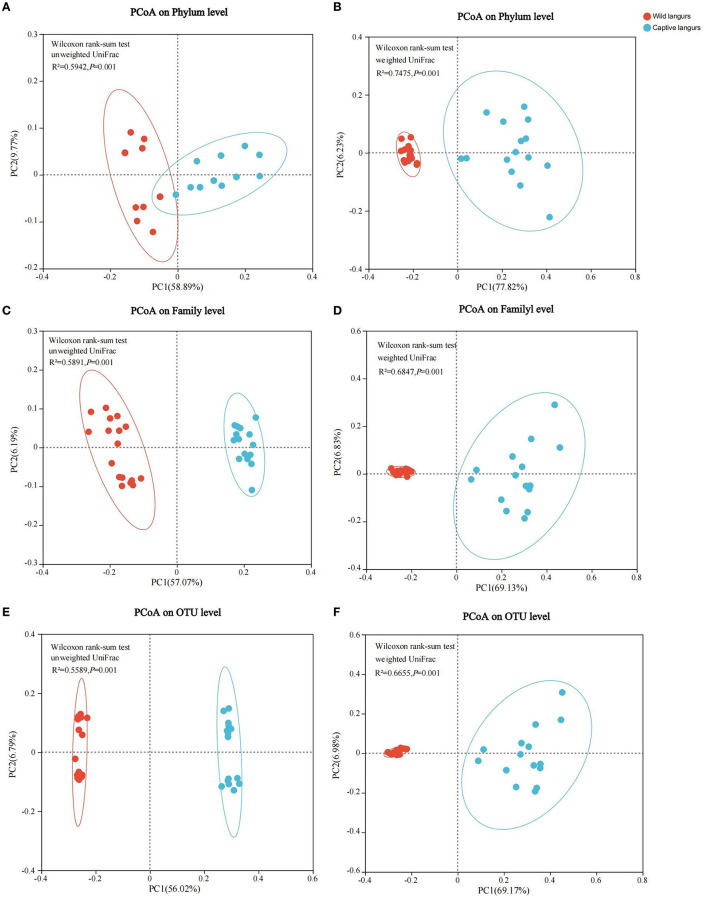
Differences in the community structure of the gut microbiota of wild and captive François' langurs at the phylum **(A, B)**, family **(C, D)**, and OTU **(E, F)** levels.

Principal coordinates analysis (PCoA) of weighted and unweighted UniFrac revealed that the captive and wild langurs gut microbiota had significant differences in the phylum and family levels (Figure 7).

### 3.4. Variations in functional profiles between captive and wild langurs gut microbiota

**Figure 8:**
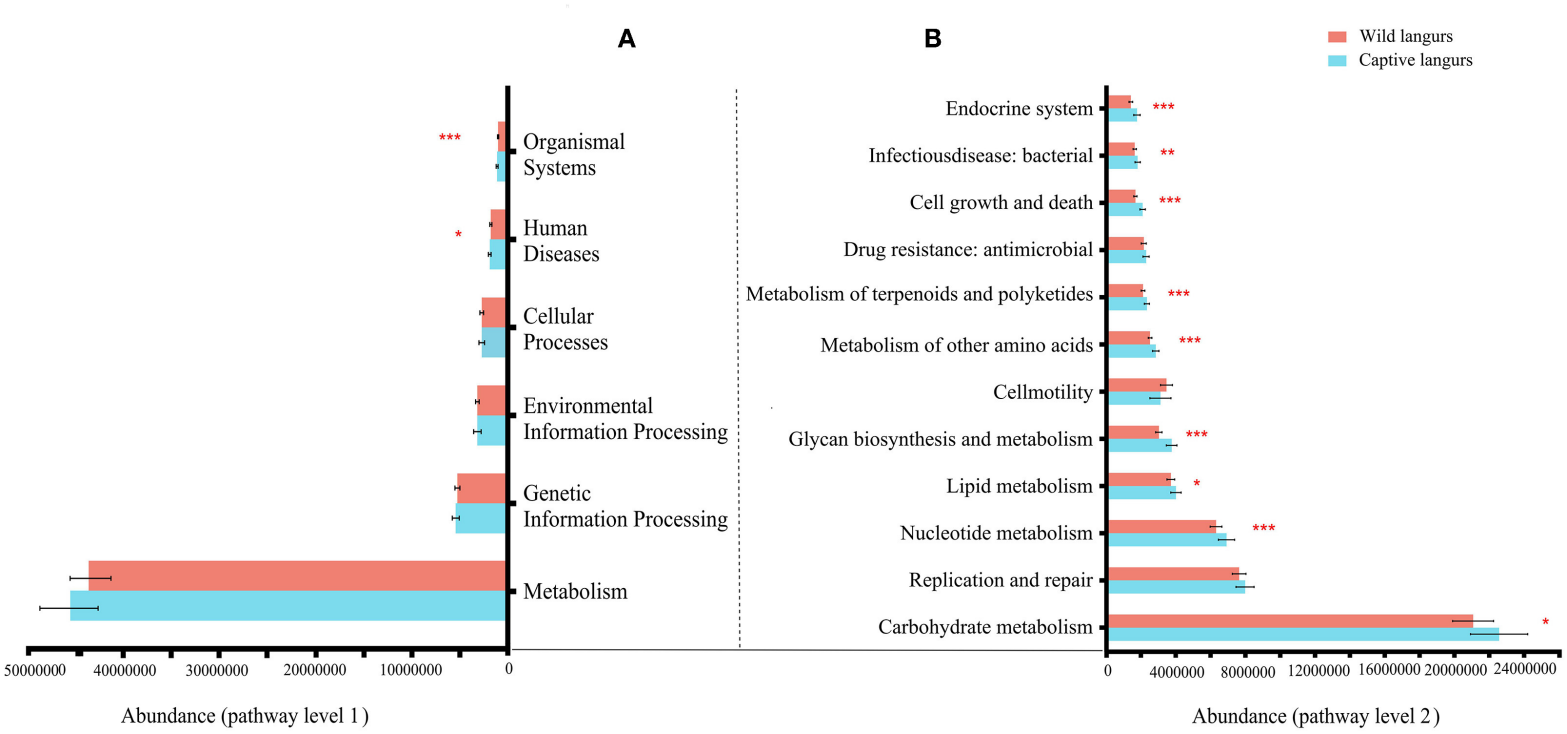
Differences in the functional profile prediction in the gut microbiota of captive and wild langurs in pathway level 1 **(A)** and pathway level 2 **(B)**. The red bar represents wild langurs and the blue one represents captive langurs. Significant difference was expressed by ^*^p < 0.05, ^**^p < 0.01, and ^***^p < 0.001.

At the KEGG pathway level 1, captive langurs enrichment was more abundant than wild. Moreover, human diseases and organismal systems significantly differed. In each channel, the enrichment in captivity was higher than that in wild (Figure 8A). Among the 46 pathways in pathway level 2, 23 showed significant differences. The 23 metabolic pathways showed significant differences, such as in nucleotide metabolism, glycan biosynthesis, metabolism, metabolism of other amino acids, metabolism of terpenoids and polyketides, cell growth and death, and the endocrine system. Except for development and regeneration, the rest showed a higher trend in captive langurs than wild (Figure 8B).

## 4. Discussion

Captive langurs have a gut microbiota predominantly characterized by the Firmicutes, Bacteroidetes, and Spirochaete, whereas wild langurs possess a gut microbiota enriched in Firmicutes, Actinobacteria, and Verrucomicrobia. Specifically, the relative abundance of bacteria related to the digestion of simple carbohydrates in captive langurs was higher than that of wild langurs, with more microbiota related to the digestion of cellulose. For example, Bacteroidetes in captive langurs were higher than in wild langurs (32.43 ± 10.00% vs. 4.82 ± 1.41%). At the family level, the overall abundance of Oscillospiraceae, Lachnospiraceae, and Christensenellaceae (included in Firmicutes) in wild langurs is higher than in captive langurs (55.48 vs. 29.21%). Thus, our study supports prediction (1).

In this study, captive langurs had a higher relative abundance of Bacteroidetes that facilitate the decomposition of polysaccharides and proteins (Fernando et al., 2010; Jami et al., 2014). Moreover, the gut microbiota of wild langurs consisted of a few high-abundance groups. The top five families accounted for more than 90% of the total abundance. This pattern could be linked to the diet of François' langurs. These langurs heavily depend on fiber-rich plant items such as leaves, fruits, and flowers (Huang et al., 2010). Particularly, they increase the consumption of mature leaves during the preferred food item-lean months (Huang et al., 2010). Compared to wild animals, captive individuals commonly consume provisioned foods rich in simple carbohydrates (Nijboer and Clauss, 2006; Chen et al., 2018). In response, wild animals increase the relative abundance of specific microbiota to facilitate the digestion of fiber-rich foods, whereas captive populations tend to increase the microbiota associated with carbohydrate-rich food digestion (Orkin et al., 2019; Sun et al., 2020). For example, Oscillospiraceae, Christensenellaceae, and Ruminococcaceae are included in Firmicutes; these bacteria ferment and break down complex fibers to provide energy and short-chain fatty acids that protect the gut and immune system (Biddle et al., 2013; Koeck et al., 2014; Reau and Suen, 2018; Waters and Ley, 2019), which are more abundant in wild individuals than captive members (Hale et al., 2019). Captive animals have more abundant microbiota associated with carbohydrate digestion, such as Prevotellaceae (Sawaswong et al., 2021). Simple carbohydrate digestion in captive animals heavily depends on *Prevotella* (Bacteroidetes), which are associated with obesity (Cuevas-Sierra et al., 2020). In the current study, captive langurs had a higher relative abundance of Prevotellaceae (16.61 ± 12.20%) than wild langurs (1.07 ± 0.86%), which is likely due to their simple carbohydrate-rich dietary composition caused by provisioned foods. An increase in the abundance of Prevotellaceae and a decrease in the abundance of Oscillospiraceae, Christensenellaceae, and Ruminococcaceae are likely linked to the consumption of plant-based carbohydrates, especially those found in fruits and high-fiber diets (Simpson and Campbell, 2015).

In addition, we found that the gut microbiota compositions of several non-human primates are similar (Table 1), which is likely due to their shared feeding habits. We find that these non-human primates adopt leaves rich in cellulose as the main foods (more than 50%), and they also have a large proportion of Firmicutes in their gut microbiota (at least more than 45%). Firmicutes degrade dietary fiber into SCFAs, which can be directly absorbed by the host (Turnbaugh et al., 2006; Sun et al., 2022). This shows that diet has a shaping effect on gut microbiota. The proportion of Bacteroidetes abundance increased with the consumption of simple carbohydrate-rich foods such as fruits, flowers, cakes, and peanuts. Bacteroidetes are characterized by fermenting carbohydrates, degrading plant-derived material, and short-chain fatty acids (Colston and Jackson, 2016) and facilitate degrading pectin in fruits and other foods (Hale et al., 2019). For example, Prevotellaceae is closely related to the proportion of fruit in animal foods (Sun et al., 2016).

**Table 1:** Gut microbiota characteristics of various primates.

**Species**	**Feeding condition**	**Dominant phyla** ^*^ **(relative abundance, %)**			**Shannon index**	**Food composition, %**						**References**
						**Leaves**	**Fruits**	**Flowers**	**Cakes**	**Bamboo shoots**	**Others**	
*Trachypithecus francoisi*	Wild	1 (76.2%)	4 (9.1%)	3 (7.7%)	4.0	91.3	6.4	–	–	–	5.2	Current study
	Wild	1 (75.4%)	3 (10.9%)	2 (9.2%)	4.2	71.0	13.2	6.3	–	–	9.5	Huang et al., 2010; Chen et al., 2020b
	Captive	1 (50.9%)	2 (32.4%)	5 (11.0%)	4.5	53.8	18.3	–	24.7	–	3.2	Current study
	Captive	1 (–)	2 (–)	5 (–)	4.3^**^	–	–	–	–	–	-	Duan et al., 2020
*T. leucocephalus*	Wild	1 (82.6%)	2 (9.2%)	4 (2.9%)	4.9^**^	94.5	3.1	0.5	–	–	1.9	Li et al., 2003; Huang et al., 2008; Que et al., 2022; Lai et al., 2023
*Rhinopithecus brelichi*	Wild	1 (46.0%)	2 (14.0%)	3 (–)	8.2^**^	60.0	–	35.0	–	–	15.0	Hale et al., 2019
*Macaca mulatta*	Wild	1 (52.0%)	2 (37.5%)	4 (3.2%)	4.1	60.5	28.0	5.1	–	–	6.4	Tang et al., 2016; Chen et al., 2020b
	Wild	1 (53.6%)	2 (33.6%)	4 (–)	4.2	–	–	–	–	–	–	Zhao et al., 2018
	Captive	2 (58.5%)	1 (32.3%)	7 (4.5%)	6.0	Monkey chow and seasonal fruit or vegetables						Cui, 2018
*M. thibetana*	Wild	1 (65.0%)	2 (26.0%)	6 (4.8%)	4.6^**^	23.3	52.5	4.5	-	14.4	5.3	Xia et al., 2021; Li et al., 2022

* 1 Firmicutes; 2 Bacteroidetes; 3 Verrucomicrobia; 4 Actinobacteria; 5 Spirochaetes; 6 Tenericutes; 7 Proteobacteria.

^−^Data not found in the literature.

** Data presented in the form of pictures and estimated according to the original literature.

The gut microbiota diversity and richness of captive langurs were higher than those of wild langurs (Figure 6), and the result of PCoA showed that the gut microbiota of wild and captive langurs were significantly different (Figure 7). In addition, at the KEGG pathway level 1, captive langurs' enrichments were more abundant than wild. Except for development and regeneration in the pathway level 2, the rest showed a higher trend in captive langurs than in wild (Figure 8). These results do not support prediction (2). Captivity has a great influence on the gut microbiota composition (Hale et al., 2019; Wang et al., 2021), which commonly causes the gut microbiota diversity to increase (Lee et al., 2019; Chen et al., 2020a) or decrease (Hale et al., 2019; Lee et al., 2019; Bornbusch et al., 2022). The dietary composition of captive animals contains more simple carbohydrates and less crude fiber and protein than wild animals (Lee et al., 2019). These are not conducive to the formation of gut microbiota in captive animals and could reduce the diversity of gut microbiota (Hale et al., 2019). However, some captive animals have a similar gut microbiota structure to wild individuals, probably because they are provisioned with natural food similar to wild animals (Martínez-Mota et al., 2020). For example, captive Guizhou snub-nosed monkeys (*Rhinopithecus brelichi*) tend to have higher abundances than their wild counterparts; moreover, their gut microbiota diversity increases in accordance with captivity (Hale et al., 2019). In this study, our sampling month was selected to be in the dry season. Previous investigations revealed that there are seasonal variations in the dietary composition of wild François' langurs (Huang et al., 2010; Zhou et al., 2018). During these months, wild langurs largely depend on mature leaves, owing to a decrease in the consumption of preferred foods such as young leaves and fruits (Huang et al., 2010). The monthly consumption of mature leaves could be attributed to the relatively simple diets of wild langurs. This pattern could be attributed to the low diversity in gut microbiota in the current study because a decrease in food items could lead to a decrease in the diversity of animal gut microbiota (Muegge et al., 2011; David et al., 2014; Sonnenburg et al., 2016; Huang et al., 2021). Captive primates largely depend on anthropogenic foods (Hale et al., 2019; Lee et al., 2019). Similarly, captive langurs in this study consumed cakes, fruits, nuts, and leaves (young leaves and mature leaves), which likely cause a higher gut microbiota diversity in captive langurs than in wild (Chen et al., 2020a). Moreover, anthropogenic food provisioning and human contact could make them susceptible to human bacteria and viruses (Wang et al., 2021), and this will humanize the gut microbiota composition (Clayton et al., 2016), likely increasing the gut microbiota diversity of the captive group (Lee et al., 2019; Bornbusch et al., 2022). Further studies on a larger temporal scale are required.

Geophagy has been considered a potential vector for the introduction of environmental microbes (Borruso et al., 2021), leading to environmental bacteria being included in the captive animals' guts. Geophagy may cause intake of some new bacteria into the gut of captive langurs, thereby leading to an increase in the gut microbiota diversity of captive langurs (Bornbusch et al., 2022). In the current study, we found the phylum of WPS-2 in the gut microbiota of François' langurs. A higher proportion of environmental microbiota could result in an increase in the gut microbiota of the provisioned langurs because they were recorded licking the grounded matrix more frequently than wild individuals (personal observation). Similar cases have been reported in rhesus macaques that contain WPS-2 in their gut microbiota associated with geophagy (Chen et al., 2020b).

## 5. Conclusion

This study preliminarily revealed differences in the gut microbiota composition between captive and wild François langurs, which is likely associated with variations in their dietary composition. The more fiber-rich foods consumed by wild langurs could increase the abundance of Firmicutes, and more simple carbohydrate-rich foods eaten by captive langurs could increase the abundance of Bacteroidetes. Our study concludes that dietary composition could be a crucial determinant in shaping the gut microbiota of langurs, highlighting the importance of captivity on the gut microbiota structure and function, and the need to consider the gut microbiota in animal provision.

## Data availability statement

Sequencing reads are available and can be found in online repositories. The name of the repository and accession number can be found below: National Center for Biotechnology Information, BioProject ID PRJNA904698, https://www.ncbi.nlm.nih.gov/bioproject/PRJNA904698. Additional datasets generated and analyzed during the study are available from the corresponding author upon reasonable request.

## Ethics statement

The animal study was reviewed and approved by the Administration Center of Guangxi Chongzuo White-headed Langur National Nature Reserve and Wuzhou Langur Breeding Center, Guangxi province, China.

## Author contributions

ZH conceived this study and revised the manuscript. YL, ZL, and JZ collected the samples and performed the experiments. FM performed the data analysis and wrote the manuscript. All authors read and approved the submitted manuscript.
