# Supplementary material for: Captivity restructures the gut microbiota of François' langurs (Trachypithecus francoisi)
Source: Front Microbiol. 2023 May 12;14:1166688. doi: 10.3389/fmicb.2023.1166688 (PMC10218129; doi:10.3389/fmicb.2023.1166688)
Supplement: Supplementary file 1 [file Table_1.DOCX]

Supplementary information 1 Showing François’ langurs gut microbiota relative abundance (Mean% ± SD%) in phylum level and the result of Wilcoxon rank-sum test.

| Taxa Name | All Sample | Wild Langurs | Captive Langurs | W Statistic | *P* Value | Corrected *P* Value |
| --- | --- | --- | --- | --- | --- | --- |
| Firmicutes | 65.33 ± 15.67 | 77.15 ± 8.37 | 51.93 ± 10.07 | 243 | < 0.001 | < 0.001 |
| Bacteroidota | 17.76 ± 15.56 | 4.82 ± 1.41 | 32.43 ± 10.00 | 0 | < 0.001 | < 0.001 |
| Spirochaetota | 5.26 ± 7.38 | 0.15 ± 0.13 | 11.04 ± 7.29 | 0 | < 0.001 | < 0.001 |
| Actinobacteriota | 4.93 ± 7.43 | 9.11 ± 8.20 | 0.20 ± 0.39 | 253 | < 0.001 | < 0.001 |
| Verrucomicrobiota | 4.16 ± 5.52 | 7.71 ± 5.52 | 0.14 ± 0.21 | 255 | < 0.001 | < 0.001 |
| Cyanobacteria | 0.86 ± 0.98 | 0.81 ± 0.59 | 0.92 ± 1.31 | 154 | 0.326 | 0.381 |
| WPS-2 | 0.57 ± 2.41 | < 0.01 | 1.21 ± 3.47 | 85 | 0.012 | 0.019 |
| Proteobacteria | 0.46 ± 0.69 | 0.13 ± 0.07 | 0.84 ± 0.87 | 22.5 | < 0.001 | < 0.001 |
| Elusimicrobiota | 0.32 ± 0.60 | < 0.01 | 0.68 ± 0.73 | 34 | < 0.001 | < 0.001 |
| Patescibacteria | 0.19 ± 0.51 | 0.07 ± 0.05 | 0.32 ± 0.74 | 187 | 0.025 | 0.037 |
| Desulfobacterota | < 0.01 | < 0.01 | < 0.01 | 0 | < 0.001 | < 0.001 |
| Campilobacterota | < 0.01 | < 0.01 | < 0.01 | 82.5 | 0.092 | 0.125 |
| Fibrobacterota | < 0.01 | < 0.01 | < 0.01 | 25.5 | < 0.001 | < 0.001 |
| unclassified_k__norank_d__Bacteria | < 0.01 | < 0.01 | < 0.01 | 23.5 | < 0.001 | < 0.001 |
| Chloroflexi | < 0.01 | < 0.01 | < 0.01 | 195 | 0.001 | 0.002 |
| Gemmatimonadota | < 0.01 | < 0.01 | < 0.01 | 135 | 0.381 | 0.381 |
| Deinococcota | < 0.01 | < 0.01 | < 0.01 | 142.5 | 0.192 | 0.243 |
| Planctomycetota | < 0.01 | < 0.01 | < 0.01 | 135 | 0.381 | 0.381 |
| Myxococcota | < 0.01 | < 0.01 | < 0.01 | 135 | 0.381 | 0.381 |
