# Supplementary material for: Captivity restructures the gut microbiota of François' langurs (Trachypithecus francoisi)
Source: Front Microbiol. 2023 May 12;14:1166688. doi: 10.3389/fmicb.2023.1166688 (PMC10218129; doi:10.3389/fmicb.2023.1166688)
Supplement: Supplementary file 2 [file Table_2.DOCX]

Supplementary information 2 Showing François’ langurs gut microbiota relative abundance (Mean% ± SD%) in family level and the result of Wilcoxon rank-sum test.

| Taxa Name | All Sample | Wild Langurs | Captive Langurs | W statistic | *P* Value | Corrected *P* Value |
| --- | --- | --- | --- | --- | --- | --- |
| Oscillospiraceae | 23.45 ± 8.82 | 30.21 ± 4.87 | 15.80 ± 5.19 | 250 | < 0.001 | < 0.001 |
| Lachnospiraceae | 9.91 ± 3.83 | 8.71 ± 3.16 | 11.28 ± 4.16 | 78 | 0.064 | 0.095 |
| Christensenellaceae | 9.79 ± 7.63 | 16.56 ± 2.66 | 2.13 ± 1.51 | 255 | < 0.001 | < 0.001 |
| Prevotellaceae | 8.36 ± 11.39 | 1.07 ± 0.86 | 16.61 ±12.20 | 1 | < 0.001 | < 0.001 |
| Spirochaetaceae | 5.25 ± 7.38 | 0.14 ± 0.12 | 11.04 ± 7.29 | 0 | < 0.001 | < 0.001 |
| Ruminococcaceae | 4.88 ± 2.43 | 4.08 ± 2.81 | 5.79 ± 1.57 | 79 | 0.070 | 0.103 |
| Eggerthellaceae | 4.40 ± 7.31 | 8.24 ± 8.37 | 0.05 ± 0.03 | 255 | < 0.001 | < 0.001 |
| Muribaculaceae | 4.06 ± 2.78 | 3.44 ± 1.54 | 4.77 ± 3.66 | 106 | 0.428 | 0.434 |
| Akkermansiaceae | 4.08 ± 5.56 | 7.68 ± 5.53 | < 0.01 | 255 | < 0.001 | < 0.001 |
| norank_o__Clostridia_UCG-014 | 3.58 ± 2.27 | 4.86 ± 2.27 | 2.13 ± 1.12 | 222 | < 0.001 | 0.001 |
| Eubacterium_coprostanoligenes_group | 3.27 ± 1.07 | 3.11 ± 1.57 | 3.46 ± 1.88 | 122 | 0.850 | 0.856 |
| Bacteroidales_RF16_group | 2.71 ± 4.10 | < 0.01 | 5.78 ± 4.27 | 9 | < 0.001 | < 0.001 |
| UCG-010 | 2.59 ± 1.45 | 2.26 ± 1.31 | 2.97 ± 1.55 | 87.5 | 0.136 | 0.186 |
| Rikenellaceae | 1.59 ± 1.81 | 0.21 ± 0.21 | 3.14 ± 1.50 | 2 | < 0.001 | < 0.001 |
| Anaerovoracaceae | 1.31 ± 1.07 | 2.18 ± 0.68 | 0.33 ± 0.18 | 255 | < 0.001 | < 0.001 |
| Acidaminococcaceae | 0.01 ± 1.32 | < 0.01 | 2.16 ± 1.10 | 0 | < 0.001 | < 0.001 |
| Monoglobaceae | 0.95 ± 0.43 | 1.26 ± 0.27 | 0.60 ± 0.27 | 243 | < 0.001 | < 0.001 |
| unclassified_c__Clostridia | 0.91 ± 0.57 | 1.08 ± 0.40 | 0.72 ± 0.67 | 196 | 0.010 | 0.020 |
| norank_o__RF39 | 0.66 ± 0.78 | 0.19 ± 0.16 | 1.19 ± 0.87 | 22.5 | < 0.001 | < 0.001 |
| Butyricicoccaceae | 0.65 ± 0.47 | 0.90 ± 0.50 | 0.37 ± 0.23 | 218 | < 0.001 | 0.002 |
| norank_o__norank_c__norank_p__WPS-2 | 0.57 ± 2.41 | < 0.01 | 1.21 ± 3.47 | 85 | 0.012 | 0.002 |
| Selenomonadaceae | 0.47 ± 0.78 | 0.02 ± 0.02 | 0.98 ± 0.91 | 16.5 | < 0.001 | < 0.001 |
| norank_o__Gastranaerophilales | 0.46 ± 0.98 | 0.06 ± 0.04 | 0.92 ± 1.31 | 17 | < 0.001 | < 0.001 |
| norank_o__Chloroplast | 0.40 ± 0.56 | 0.75 ± 0.57 | < 0.01 | 255 | < 0.001 | < 0.001 |
| norank_o__Clostridia_vadinBB60_group | 0.34 ± 0.35 | 0.07 ± 0.04 | 0.56 ± 0.28 | 0 | < 0.001 | < 0.001 |
| Elusimicrobiaceae | 0.32 ± 0.60 | < 0.01 | 0.68 ± 0.73 | 34 | < 0.001 | < 0.001 |
| unclassified_o__Bacteroidales | 0.32 ± 0.88 | 0.01 ± 0.01 | 0.67 ± 1.21 | 56 | 0.006 | 0.013 |
| p-2534-18B5_gut_group | 0.29 ± 0.57 | 0.03 ± 0.02 | 0.58 ± 0.74 | 82.5 | 0.092 | 0.134 |
| unclassified_o__Oscillospirales | < 0.01 | < 0.01 | < 0.01 | 156 | 0.290 | 0.345 |
| norank_o__Izemoplasmatales | 0.23 ± 0.40 | 0.03 ± 0.02 | 0.45 ± 0.50 | 65 | 0.019 | 0.034 |
| Anaerofustaceae |  |  |  | 251 | < 0.001 | < 0.001 |
| Peptococcaceae | 0.21 ± 0.43 | 0.35 ± 0.56 | 0.06 ± 0.40 | 245.5 | < 0.001 | < 0.001 |
| Succinivibrionaceae | 0.19 ± 0.58 | < 0.01 | 0.40 ±0.80 | 76.5 | 0.005 | 0.011 |
| Saccharimonadaceae | 0.18 ± 0.51 | 0.06 ± 0.05 | 0.32 ± 0.74 | 187 | 0.024 | 0.042 |
| Tannerellaceae | 0.18 ± 0.28 | < 0.01 | 0.38 ± 0.29 | 0 | < 0.001 | < 0.001 |
| norank_o__Rhodospirillales | < 0.01 | < 0.01 | < 0.01 | 1.5 | < 0.001 | < 0.001 |
| F082 | 0.14 ± 0.73 | < 0.01 | 0.30 ± 1.06 | 110.5 | 0.138 | 0.186 |
| Peptostreptococcaceae | < 0.01 | < 0.01 | < 0.01 | 247.5 | < 0.001 | < 0.001 |
| Micrococcaceae | 0.14 ± 0.38 | 0.27 ±0.49 | < 0.01 | 225 | < 0.001 | < 0.001 |
| Atopobiaceae | < 0.01 | < 0.01 | < 0.01 | 255 | < 0.001 | < 0.001 |
| Erysipelotrichaceae | < 0.01 | < 0.01 | < 0.01 | 2.5 | < 0.001 | < 0.001 |
| norank_o__WCHB1-41 | < 0.01 | < 0.01 | < 0.01 | 74 | 0.045 | 0.075 |
| Desulfovibrionaceae | < 0.01 | < 0.01 | < 0.01 | 0 | < 0.001 | < 0.001 |
| norank_o__norank_c__Clostridia | < 0.01 | < 0.01 | < 0.01 | 198 | 0.008 | 0.016 |
| Coriobacteriaceae | 0.06 ± 0.27 |  | 0.12 ± 0.39 | 8.5 | < 0.001 | < 0.001 |
| Erysipelatoclostridiaceae | < 0.01 | < 0.01 | < 0.01 | 0 | < 0.001 | < 0.001 |
| norank_o__Coriobacteriales | < 0.01 | < 0.01 | < 0.01 | 237 | < 0.001 | < 0.001 |
| Bacteroidaceae | < 0.01 | < 0.01 | < 0.01 | 9.5 | < 0.001 | < 0.001 |
| unclassified_p__Firmicutes | < 0.01 | < 0.01 | < 0.01 | 41 | < 0.001 | 0.002 |
| norank_o__Oscillospirales | < 0.01 | < 0.01 | < 0.01 | 243.5 | < 0.001 | < 0.001 |
| Sutterellaceae | < 0.01 | < 0.01 | < 0.01 | 0 | < 0.001 | < 0.001 |
| Corynebacteriaceae | < 0.01 | < 0.01 | < 0.01 | 195 | 0.001 | 0.003 |
| Dermatophilaceae | < 0.01 | < 0.01 | < 0.01 | 187.5 | 0.003 | 0.007 |
| Clostridiaceae | < 0.01 | < 0.01 | < 0.01 | 64.5 | 0.018 | 0.032 |
| Fibrobacteraceae | < 0.01 | < 0.01 | < 0.01 | 25.5 | < 0.001 | < 0.001 |
| Campylobacteraceae | < 0.01 | < 0.01 | < 0.01 | 123 | 0.880 | 0.880 |
| Flavobacteriaceae | < 0.01 | < 0.01 | < 0.01 | 59.5 | < 0.001 | 0.002 |
| unclassified_o__Coriobacteriales | < 0.01 | < 0.01 | < 0.01 | 255 | < 0.001 | < 0.001 |
| norank_o__Bacteroidales | < 0.01 | < 0.01 | < 0.01 | 75 | 0.009 | 0.019 |
| Defluviitaleaceae | < 0.01 | < 0.01 | < 0.01 | 0.5 | < 0.001 | < 0.001 |
| Coriobacteriales_Incertae_Sedis | < 0.01 | < 0.01 | < 0.01 | 255 | < 0.001 | < 0.001 |
| Oxalobacteraceae | < 0.01 | < 0.01 | < 0.01 | 164 | 0.171 | 0.227 |
| Eubacteriaceae | < 0.01 | < 0.01 | < 0.01 | 215 | < 0.001 | 0.001 |
| unclassified_k__norank_d__Bacteria | < 0.01 | < 0.01 | < 0.01 | 23.5 | < 0.001 | < 0.001 |
| unclassified_c__Bacteroidia | < 0.01 | < 0.01 | < 0.01 | 240 | < 0.001 | < 0.001 |
| Carnobacteriaceae | < 0.01 | < 0.01 | < 0.01 | 187.5 | 0.003 | 0.007 |
| Marinifilaceae | < 0.01 | < 0.01 | < 0.01 | 23.5 | < 0.001 | < 0.001 |
| norank_o__Bradymonadales | < 0.01 | < 0.01 | < 0.01 | 85 | 0.012 | 0.022 |
| Burkholderiaceae | < 0.01 | < 0.01 | < 0.01 | 229.5 | < 0.001 | < 0.001 |
| Brachyspiraceae | < 0.01 | < 0.01 | < 0.01 | 226.5 | < 0.001 | < 0.001 |
| norank_o__Veillonellales-Selenomonadales | < 0.01 | < 0.01 | < 0.01 | 160 | 0.212 | 0.256 |
| Helicobacteraceae | < 0.01 | < 0.01 | < 0.01 | 76.5 | 0.005 | 0.011 |
| unclassified_o__Micrococcales | < 0.01 | < 0.01 | < 0.01 | 195 | 0.001 | 0.003 |
| unclassified_o__Lachnospirales | < 0.01 | < 0.01 | < 0.01 | 198 | 0.004 | 0.009 |
| Paracaedibacteraceae | < 0.01 | < 0.01 | < 0.01 | 255 | < 0.001 | < 0.001 |
| unclassified_o__Veillonellales-Selenomonadales | < 0.01 | < 0.01 | < 0.01 | 43 | < 0.001 | 0.001 |
| unclassified_o__Rickettsiales | < 0.01 | < 0.01 | < 0.01 | 57.5 | 0.004 | 0.009 |
| JG30-KF-CM45 | < 0.01 | < 0.01 | < 0.01 | 195 | 0.001 | 0.003 |
| Propionibacteriaceae | < 0.01 | < 0.01 | < 0.01 | 195 | 0.001 | 0.003 |
| Rhodobacteraceae | < 0.01 | < 0.01 | < 0.01 | 210 | < 0.001 | < 0.001 |
| Mitochondria | < 0.01 | < 0.01 | < 0.01 | 225 | < 0.001 | < 0.001 |
| norank_o__norank_c__norank_p__Firmicutes | < 0.01 | < 0.01 | < 0.01 | 110.5 | 0.138 | 0.186 |
| unclassified_o__Burkholderiales | < 0.01 | < 0.01 | < 0.01 | 96 | 0.140 | 0.187 |
| Sphingomonadaceae | < 0.01 | < 0.01 | < 0.01 | 217.5 | < 0.001 | < 0.001 |
| norank_o__Saccharimonadales | < 0.01 | < 0.01 | < 0.01 | 215 | < 0.001 | 0.002 |
| Enterobacteriaceae | < 0.01 | < 0.01 | < 0.01 | 110 | 0.264 | 0.317 |
| Oligosphaeraceae | < 0.01 | < 0.01 | < 0.01 | 85 | 0.012 | 0.022 |
| Dermabacteraceae | < 0.01 | < 0.01 | < 0.01 | 195 | 0.001 | 0.003 |
| Nocardiaceae | < 0.01 | < 0.01 | < 0.01 | 215 | < 0.001 | 0.001 |
| Lactobacillaceae | < 0.01 | < 0.01 | < 0.01 | 216 | < 0.001 | 0.001 |
| Acholeplasmataceae | < 0.01 | < 0.01 | < 0.01 | 119 | 0.316 | 0.371 |
| Clostridium_methylpentosum_group | < 0.01 | < 0.01 | < 0.01 | 196.5 | 0.005 | 0.010 |
| Staphylococcaceae | < 0.01 | < 0.01 | < 0.01 | 142.5 | 0.192 | 0.234 |
| unclassified_p__Proteobacteria | < 0.01 | < 0.01 | < 0.01 | 225 | < 0.001 | < 0.001 |
| Microbacteriaceae | < 0.01 | < 0.01 | < 0.01 | 187.5 | 0.003 | 0.007 |
| Devosiaceae | < 0.01 | < 0.01 | < 0.01 | 195 | 0.001 | 0.003 |
| Hungateiclostridiaceae | < 0.01 | < 0.01 | < 0.01 | 110.5 | 0.138 | 0.186 |
| Rhizobiaceae | < 0.01 | < 0.01 | < 0.01 | 195 | 0.001 | 0.003 |
| Caulobacteraceae | < 0.01 | < 0.01 | < 0.01 | 180 | 0.007 | 0.013 |
| Brevibacteriaceae | < 0.01 | < 0.01 | < 0.01 | 157.5 | 0.053 | 0.081 |
| Intrasporangiaceae | < 0.01 | < 0.01 | < 0.01 | 142.5 | 0.341 | 0.391 |
| Bogoriellaceae | < 0.01 | < 0.01 | < 0.01 | 180 | 0.007 | 0.133 |
| Nocardioidaceae | < 0.01 | < 0.01 | < 0.01 | 165 | 0.027 | 0.046 |
| Veillonellaceae | < 0.01 | < 0.01 | < 0.01 | 188.5 | 0.005 | 0.011 |
| vadinBE97 | < 0.01 | < 0.01 | < 0.01 | 111.5 | 0.418 | 0.426 |
| Acetobacteraceae | < 0.01 | < 0.01 | < 0.01 | 165 | 0.027 | 0.046 |
| Beijerinckiaceae | < 0.01 | < 0.01 | < 0.01 | 187.5 | 0.003 | 0.007 |
| Xanthomonadaceae | < 0.01 | < 0.01 | < 0.01 | 157.5 | 0.053 | 0.081 |
| Pseudonocardiaceae | < 0.01 | < 0.01 | < 0.01 | 165 | 0.027 | 0.046 |
| unclassified_o__Rhodospirillales | < 0.01 | < 0.01 | < 0.01 | 102 | 0.062 | 0.093 |
| Bacillaceae | < 0.01 | < 0.01 | < 0.01 | 172.5 | 0.013 | 0.024 |
| Comamonadaceae | < 0.01 | < 0.01 | < 0.01 | 157.5 | 0.053 | 0.081 |
| Planococcaceae | < 0.01 | < 0.01 | < 0.01 | 135 | 0.381 | 0.391 |
| Xanthobacteraceae | < 0.01 | < 0.01 | < 0.01 | 157.5 | 0.053 | 0.081 |
| Moraxellaceae | < 0.01 | < 0.01 | < 0.01 | 142.5 | 0.192 | 0.234 |
| Cellvibrionaceae | < 0.01 | < 0.01 | < 0.01 | 142.5 | 0.192 | 0.234 |
| Micromonosporaceae | < 0.01 | < 0.01 | < 0.01 | 157.5 | 0.052 | 0.081 |
| Puniceicoccaceae | < 0.01 | < 0.01 | < 0.01 | 85 | 0.012 | 0.022 |
| norank_o__Gaiellales | < 0.01 | < 0.01 | < 0.01 | 135 | 0.381 | 0.391 |
| Dietziaceae | < 0.01 | < 0.01 | < 0.01 | 150 | 0.101 | 0.141 |
| unclassified_c__Bacilli | < 0.01 | < 0.01 | < 0.01 | 157.5 | 0.052 | 0.081 |
| Thermomonosporaceae | < 0.01 | < 0.01 | < 0.01 | 150 | 0.100 | 0.141 |
| Longimicrobiaceae | < 0.01 | < 0.01 | < 0.01 | 135 | 0.381 | 0.391 |
| Chroococcidiopsaceae | < 0.01 | < 0.01 | < 0.01 | 142.5 | 0.192 | 0.234 |
| unclassified_c__Alphaproteobacteria | < 0.01 | < 0.01 | < 0.01 | 119 | 0.316 | 0.371 |
| Cyclobacteriaceae | < 0.01 | < 0.01 | < 0.01 | 142.5 | 0.192 | 0.234 |
| Sphingobacteriaceae | < 0.01 | < 0.01 | < 0.01 | 135 | 0.381 | 0.391 |
| AKYG1722 | < 0.01 | < 0.01 | < 0.01 | 135 | 0.381 | 0.391 |
| Kineosporiaceae | < 0.01 | < 0.01 | < 0.01 | 135 | 0.381 | 0.391 |
| Porphyromonadaceae | < 0.01 | < 0.01 | < 0.01 | 142.5 | 0.192 | 0.234 |
| A4b | < 0.01 | < 0.01 | < 0.01 | 150 | 0.100 | 0.141 |
| Aerococcaceae | < 0.01 | < 0.01 | < 0.01 | 142.5 | 0.192 | 0.234 |
| Ilumatobacteraceae | < 0.01 | < 0.01 | < 0.01 | 157.5 | 0.052 | 0.081 |
| Victivallaceae | < 0.01 | < 0.01 | < 0.01 | 102 | 0.062 | 0.093 |
| Streptococcaceae | < 0.01 | < 0.01 | < 0.01 | 102 | 0.062 | 0.093 |
| Microtrichaceae | < 0.01 | < 0.01 | < 0.01 | 135 | 0.381 | 0.391 |
| Spirosomaceae | < 0.01 | < 0.01 | < 0.01 | 150 | 0.100 | 0.141 |
| Solirubrobacteraceae | < 0.01 | < 0.01 | < 0.01 | 142.5 | 0.192 | 0.234 |
| Nocardiopsaceae | < 0.01 | < 0.01 | < 0.01 | 135 | 0.381 | 0.391 |
| Trueperaceae | < 0.01 | < 0.01 | < 0.01 | 142.5 | 0.192 | 0.234 |
| Steroidobacteraceae | < 0.01 | < 0.01 | < 0.01 | 135 | 0.381 | 0.391 |
| Bifidobacteriaceae | < 0.01 | < 0.01 | < 0.01 | 135 | 0.381 | 0.391 |
| unclassified_o__Cyanobacteriales | < 0.01 | < 0.01 | < 0.01 | 142.5 | 0.192 | 0.234 |
| Saprospiraceae | < 0.01 | < 0.01 | < 0.01 | 135 | 0.381 | 0.391 |
| norank_o__Peptostreptococcales-Tissierellales | < 0.01 | < 0.01 | < 0.01 | 135 | 0.381 | 0.391 |
| Reyranellaceae | < 0.01 | < 0.01 | < 0.01 | 135 | 0.381 | 0.391 |
| norank_o__Actinomarinales | < 0.01 | < 0.01 | < 0.01 | 135 | 0.381 | 0.391 |
| Sandaracinaceae | < 0.01 | < 0.01 | < 0.01 | 135 | 0.381 | 0.391 |
| TRA3-20 | < 0.01 | < 0.01 | < 0.01 | 135 | 0.381 | 0.391 |
| Pirellulaceae | < 0.01 | < 0.01 | < 0.01 | 135 | 0.381 | 0.391 |
