# Supplementary material for: Captivity restructures the gut microbiota of François' langurs (Trachypithecus francoisi)
Source: Front Microbiol. 2023 May 12;14:1166688. doi: 10.3389/fmicb.2023.1166688 (PMC10218129; doi:10.3389/fmicb.2023.1166688)

Supplementary information 3 Community composition of gut microbiota in François’ langurs at the phylum (A), family (B) and OTU (C).

A


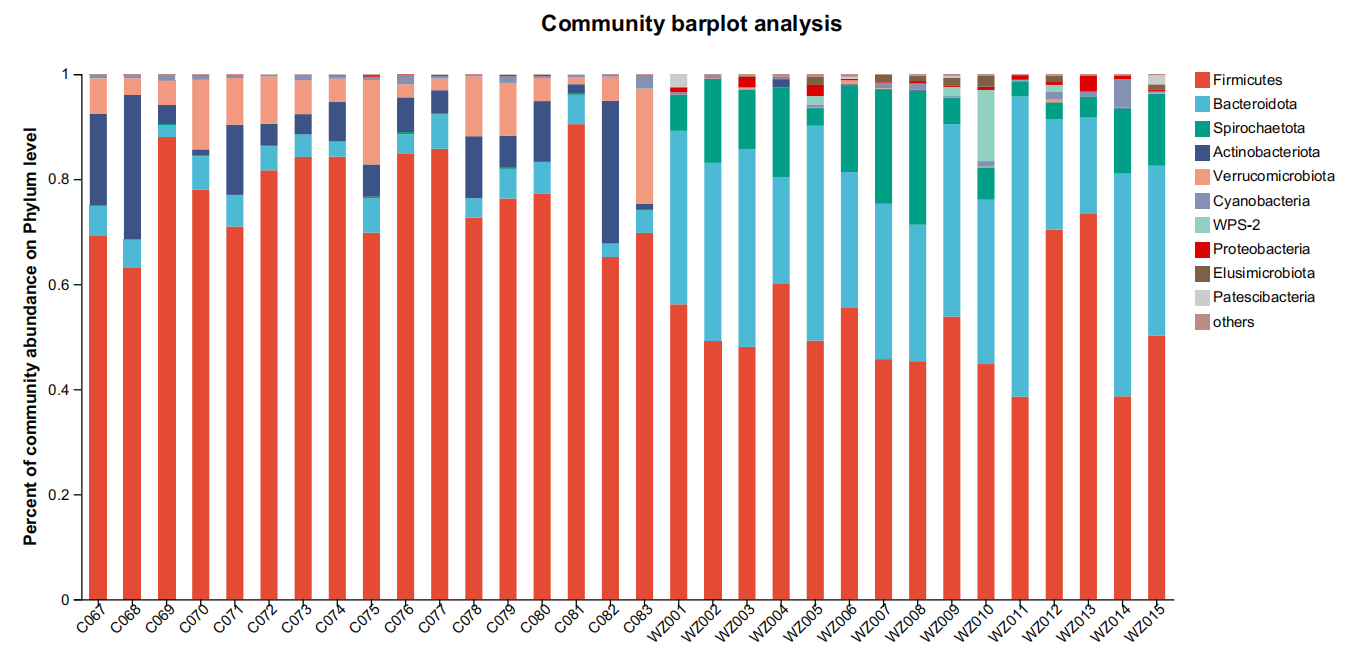


B


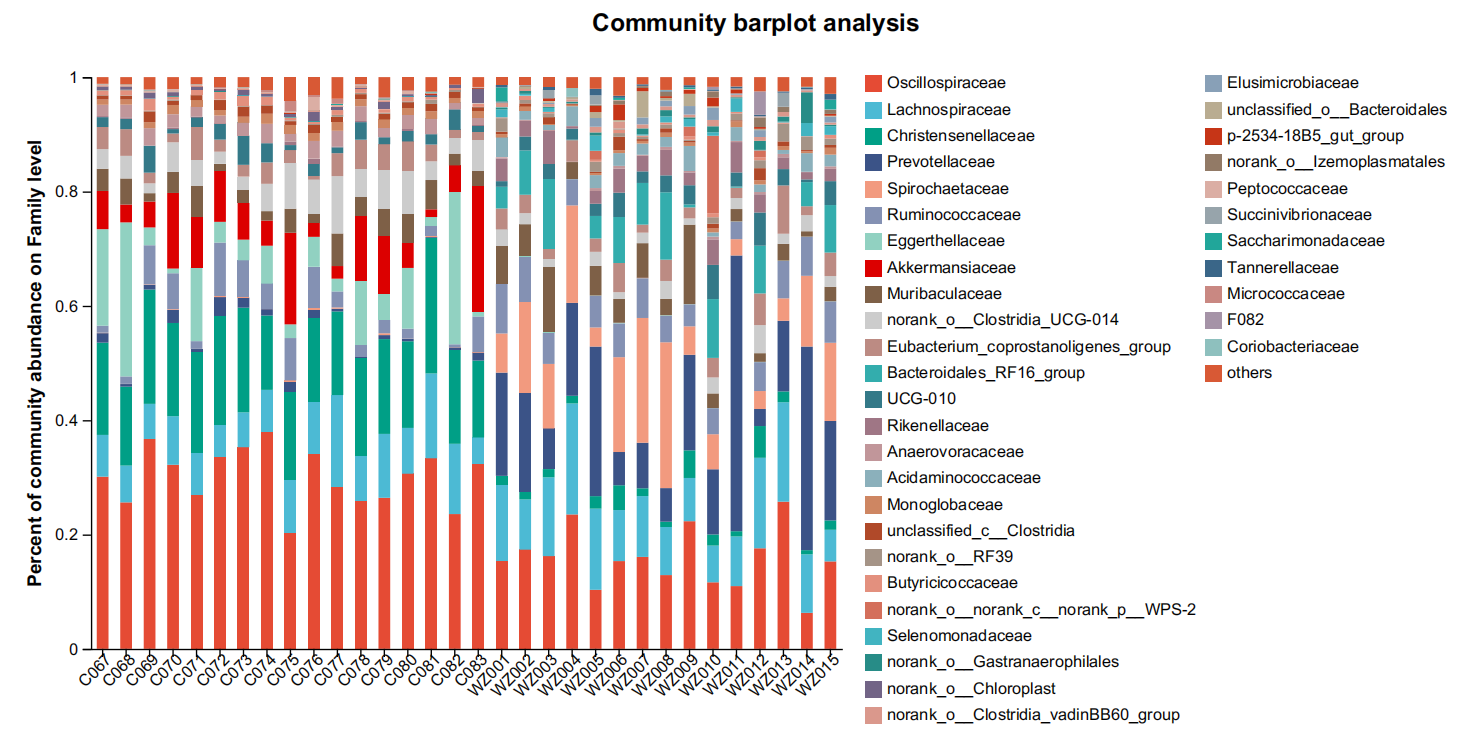


C


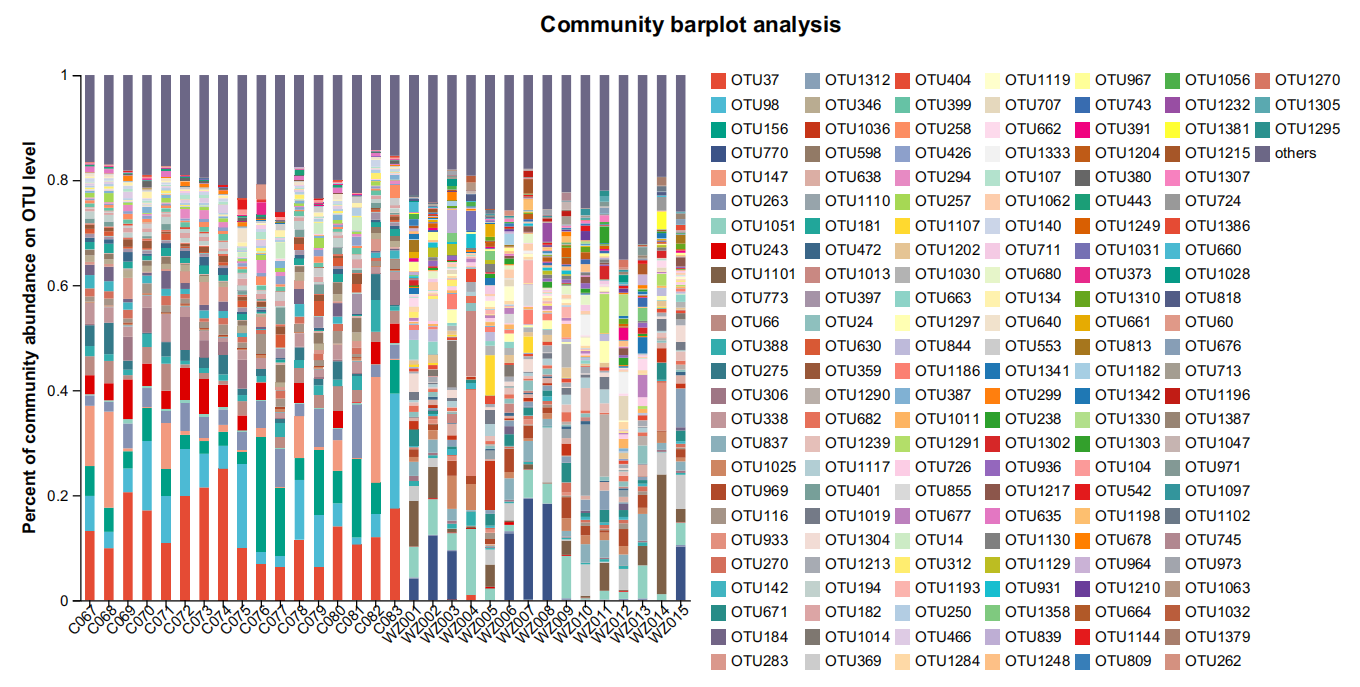

Supplement: Supplementary file 3 [file Table_3.DOCX]
